# The Institutional Worker

**Published:** 1912-02-17

**Authors:** 


THE
### INSTITUTIONAL WORKER
### Being a Special Supplement to "The Hospital"

### Editor's Notices.

**Contributions:**

Contributions should be written, or preferably typed, on one side of the paper only, and all articles sent in are accepted upon the distinct understanding that they are forwarded to The Hospital only.

The Editor cannot undertake to return MSS. not used, but when a stamped directed envelope is enclosed the MSS. may be returned if a special request is made.

Accepted articles and paragraphs of news will be paid for after publication at the scale rate.

**Address:**

To prevent delay all contributions and letters on editorial business must be addressed exclusively to the Editor, "The Hospital," 29 Southampton Street, Strand, London, W.C. It is important that this regulation shall be strictly observed.

**Correspondence:**

Correspondence on all subjects is invited. The name and address of correspondents must be given as a guarantee of good faith, but not necessarily for publication.

**Special Articles:**

Special articles are invited, and questions, inquiries, and paragraphs upon all matters relating to the work, administration, and management of general, special, mental, fever, cottage and convalescent hospitals, sanatoria, homes, institutions, societies and organisations for the treatment or care of the sick, injured and dependents of all classes. Every matter affecting the interests and welfare of the staff of all grades working in these institutions will receive special consideration.

**Administrative Medicine, Research and Items of News:**

Special terms will be paid for approved articles on subjects in this wide field by experts who have made some section of it their special study and interest. We wish to give prominence to every movement tending to advance accuracy of diagnosis, efficiency in treatment, and the development of modern methods to eradicate disease and promote the welfare of the suffering.

**A Bureau of Information:**

We invite inquiries and applications for information and help in providing for that numerous class of sufferers who require special care or house-room which they cannot obtain in their own homes or secure for themselves. We cannot, however, prescribe or recommend practitioners.

**Books for Review:**

Publishers are particularly requested to send advance proofs of any new books of importance whenever possible, as the Editor has made arrangements to publish immediate reviews on a new plan.

**Photographs, Plans, Blocks, and Illustrations:**

It is requested that wherever possible MSS. may be accompanied by illustrations in any of the above forms. The name of the sender and of the article to which it belongs should be written on the back of each photograph, drawing, or block for purposes of identification.

**Local Papers:**

Newspapers containing reports or news paragraphs should be marked and addressed to the Sub-Editor.

**The Coupon System:**

A coupon will be found at the bottom of the third inside page of the cover each week. This coupon must be attached to every question or inquiry to which an answer is desired in The Hospital.

### Manager's Notices.

Letters relating to the publication, sale, and advertising departments of The Hospital must be addressed to the Manager, "The Hospital," The Hospital Building, 28 and 29 Southampton Street, Strand, London, W.C.

Subscriptions which may begin at any time, are payable in advance. Cheques and Post-Office orders, crossed London County and Westminster Bank, Covent Garden branch, should be made payable to the Manager of The Hospital and addressed to him at The Hospital Building, 28 and 29 Southampton Street, Strand, London, W.C.

**Advertisements:**

All advertisements for The Hospital must reach the Manager not later than Wednesday morning in each week. For scale of charges see pages v and 2.

**Institution Authorities.**

The economical management of an Institution depends very greatly upon purchasing supplies in the cheapest market.

It is often impossible locally to secure the highest quality at the lowest prices.

In consequence, a section will be reserved in this part of the paper for announcements of Institutions inviting Tenders for Contracts.

This will enable Authorities of Institutions to secure tenders for supplies from all over the country, and ensure purchases at the lowest possible prices consistent with quality.

The charge for Tenders is 6s. per inch, and for this small outlay many pounds may be saved on supplies of all descriptions.

Special Rates will be quoted for those institutions which agree to send all their vacancy, contract, and public notices to The Hospital each year, so as to make it a file of such announcements for ready reference.

**Classified Advertisements:**

All small advertisements will be classified, for this section of the paper is widely read and of special interest. We wish to encourage those wanting appointments who are attracted to institutional work, and therefore offer them the reduced rate of *twenty-one words and under for* 1s., and *for every ten and part of ten words beyond*, 6*d*.

This form may be used for small prepaid advertisements, and when filled up should be posted to the

*Manager*, The Hospital,
28 and 29 Southampton Street,
Strand, W.C.

Appointments Wanted, 21 words ... 1s. 0d.
Appointments Vacant, 21 words ... 2s. 6d.

### OFFICIAL APPOINTMENTS VACANT.

### Poor-Law Vacancies.

ASSISTANT LAUNDRESS (£25), Ecclesall Bierlow Union. Mr. J. E. Moulding, C. to G., The Edge, Sheffield. Feb. 21.

ASSISTANT MALE ATTENDANT (£25), Ashton-under-Lyne Union. Mr. G. H. Partington, C. to G., Ashton-under-Lyne. Feb. 20.

ASSISTANT MALE ATTENDANT (£35), Ecclesall Bierlow Union. Mr. J. E. Moulding, C. to G., The Edge, Sheffield. Feb. 21.

ASSISTANT MATRON (£30), Plymouth Guardians. Mr. W. H. Davy, C. to G., Plymouth. Feb. 20.

ASSISTANT NURSE (£25), Wokingham Union. Mr. J. F. Sargeant, C. to G., Wokingham. Feb. 19.

ASSISTANT NURSE (£16), Ashbourne Union. Mr. W. R. Holland, C. to G., Ashbourne. Feb. 22.

ASSISTANT NURSE (£22 10s.), Ware Union. Mr. G. H. Gisby, C. to G., Ware, Herts. Mar. 4.

ASSISTANT NURSE (£27 10s.), Alcester Union. G. Thomas, C. to G., Alcester.

ASSISTANT NURSE (£23), Rugby Union J. W. Pendred, C. to G., Union Office, Rugby. Feb. 22.

ASSISTANT NURSE (£25), W. S. V. Cullerne, C. to G., Commercial Road, Guildford. March 1.

ASSISTANT SCHOOLMASTER (£75), Holme Court Industrial School. Mr. S. Eslick, Clerk to the Managers, Isleworth, W. Feb. 22.

BOYS' ATTENDANT (£35), Edmonton Union. Mr. F. Shelton, C. to G., Lower Tottenham, N. Feb. 17.

CASE-PAPER CLERK (£100), Shoreditch Guardians. Mr. R. Clay, C. to G., 213 Kingsland Road, N.E. Feb. 17.

CHARGE NURSE (£28), Holborn Union. Matron of the Workhouse, 1A Shepherdess Walk, N.

CHARGE NURSE (£36), Paddington Guardians. Mr. H. F. Aveling, C. to G., 313-319 Harrow Road, W. Feb. 21.

CHARGE NURSE (£30), St. John, Hampstead Guardians. H. W. Preston, C. to G., New End, Hampstead. Feb. 27.

CLERK OF WORKS (Infirmary) (£2 2s. weekly), Elham Union. Mr. E. Lovick, C. to G., 29 Bouverie Square, Folkestone. Feb. 27.

CLERKS (two at £80 per annum and four, temporary, 24s. weekly), Liverpool Guardians. Mr. G. W. Coster, Vestry Clerk, Liverpool. Feb. 17.

COOK (£25), Frome Union. Mr. W. R. Kent, C. to G., Frome. Feb. 19.

FEMALE ATTENDANT (£20), Southwark Union. Mr. G. Wood, C. to G., Ufford Street, Blackfriars Road, S.E. Feb. 20.

FEMALE ATTENDANT (£25), Lambeth Guardians. Matron of the Parish School, Elder Road, West Norwood, S.E.

FEMALE ATTENDANT (£23), Islington Guardians. Master of the Workhouse, Cornwallis Road, Upper Holloway, N.

FEMALE BATH ATTENDANT, ETC. (£25), Islington Guardians. Master of the Workhouse, Cornwallis Road, Upper Holloway, N.

FEMALE CHARGE ATTENDANT (£30), Portsmouth Guardians. Mr. E. H. Mitchell, C. to G., Portsmouth. Feb. 19.

FEMALE COOK (£30), Farnham and Hartley Wintney Schools. Superintendent of the Schools, Crondall, Hants.

FEMALE SICK ATTENDANT (£28), Edmonton Union. Mr. F. Shelton, C. to G., Lower Tottenham, N. Feb. 17.

FOSTER-MOTHER (£28), Edmonton Union. Mr. F. Shelton, C. to G., Lower Tottenham, N. Feb. 17.

GIRLS' ATTENDANT (£30), Edmonton Union. Mr. F. Shelton, C. to G., Lower Tottenham, N. Feb. 17.

HEAD NURSE (£30), Falmouth Union. Mr. R. N. Rogers, C. to G., Union Offices, Falmouth. Feb. 20.

HEAD NURSE (£30), Redruth Union. Mr. T. Peter, C. to G., Redruth. Feb. 29.

JUNIOR ASSISTANT NURSE (£15), Malling Union. F. J. Allison, C. to G., West Malling, Kent.

LABOUR MASTERS (2) (£30 each), Islington Guardians. Master of the Workhouse, Cornwallis Road, Upper Holloway, N.

LADY CROSS VISITOR (£75), Stoke-upon-Trent Union. H. G. Poole, Acting C. to G., Union Offices, Stoke-on-Trent.

LAUNDRESS (£30), Wigan Union. Mr. H. G. Ackerley, C. to G., Wigan. Feb. 24.

